# Ecological relevance in honeybee pesticide risk assessment: developing context-dependent scenarios to manage uncertainty

**DOI:** 10.3389/fphys.2013.00062

**Published:** 2013-04-04

**Authors:** Mickaël Henry, Axel Decourtye

**Affiliations:** ^1^INRA, UR406 Abeilles et Environnement, Site AgroparcAvignon, France; ^2^UMT Protection des Abeilles dans l'Environnement, Site AgroparcAvignon, France; ^3^Association de Coordination Technique Agricole, Site AgroparcAvignon, France

We reported 1 year ago (Henry et al., 2012a) that a sublethal dose of thiamethoxam, a neonicotinoid pesticide used on some common flowering crops, reduces the ability of exposed honeybees to find their way back to the colony. More recently, Guez (2013) have raised concerns about the relevance of our study design. The obvious intention of the author was to discredit our study, both by seeking after inconsistencies in calculations and gathering new arguments against the experimental dose of thiamethoxam. In both case, we would like to carry out major rectifications of his statements. We fully agree that there is still place for improvement in the way one may estimate pesticide exposure levels in honeybees, and Guez (2013) brought interesting thoughts in that direction. However, this should be done with respect to the recent advance on that topic (ANSES, 2012; Cresswell and Thompson, 2012; EFSA, 2012a,b, 2013; Henry et al., 2012b) and with respect to the need for hierarchizing the sources of uncertainty. Herein, we show that the overestimation issue raised by Guez has already been addressed before and with an even greater magnitude of uncertainty. But at the heart of the debate is the way to deal with uncertainty in estimates of honeybee field exposure to pesticides. In that respect, we further underline (1) the need to properly hierarchize uncertainties using conventional ecological scaling approaches and (2) the need to investigate *context-dependent* exposure scenarios, whereby *worse-case* situations are explicitly linked with their spatial or temporal occurrence likelihood.

Our main concern about Guez's comment is that the conclusions on the peer review of the risk assessment for bees, published consecutively to our study, were basically ignored (ANSES, 2012; EFSA, 2012a,b, 2013). The updated expertise and field measurements of nectar residues intake returned exposure levels of 0.10–0.33 ng.bee^−1^.day^−1^ (ANSES, 2012), 0.184–5.888 ng.bee^−1^.day^−1^ (EFSA, 2012a) and 0.553–2.903 ng.bee^−1^.day^−1^ (EFSA, 2013) for winter oilseed rape treated with thiamethoxam. Based on these results, one must admit that uncertainties in field exposure levels are so high that they should be the prime focus of risk assessors interested in refining honeybee exposure scenarios. Depending on the considered scenario, our 1.34 ng dose may actually overestimate by a factor of 13.4 or underestimate by a factor of 4.4 the real field exposure level. This actually overwhelms the uncertainty levels raised by Guez due to temporal variations in sugar content (<6-fold overestimations, Figure 1A in Guez, 2013) and due to spatial variations in daylight time available for foraging (Figure 1B in Guez, 2013). Although Guez made valuable efforts to challenge our study design, there is a higher level of uncertainty to deal with first.

## Hierarchizing uncertainties

This merely illustrates the need to conveniently hierarchize uncertainty levels in pesticide risk assessment. Each parameter used for the field exposure assessment (e.g., sugar concentration, flight time, pesticide residuals in nectar; Rortais et al., 2005) is associated with an intrinsic uncertainty, denoted by an upper and a lower bound. The successive multiplication of uncertainty bounds considerably increases the overall uncertainty. As the pesticide risk assessment debate slides from pure toxicology to ecotoxicology and behavioral ecology, this problem is being exacerbated by the accumulation of ecological parameters, each piling up an additional uncertainty. Guez (2013) offers a typical counterexample of uncertainty ranking. His comment first expands on why we have reported daily exposure estimates of 0.17–2.3 ng instead of an expected 0.197–2.375 ng. The discussion turns around this deviation equivalent to 2.0–5.6% of our experimental dose (1.34 ng), whereas we learn in the next section that our dose may actually overestimate real exposure by as much as 140–600% (Figure 1A in Guez, 2013). We feel that two orders of magnitude should appeal for a better discernment of which concern to put forward. Notwithstanding, would some readers be interested, the range deviation results from successive rounding at the different stages of the calculation. We have obviously rounded off at coarser levels than Guez, resulting in a different level of resolution (±0.01–0.1 ng, not 0.001 ng). We feel it was reasonable not to go into thinner levels of resolution given the high uncertainty inherent to all the involved parameters. Likewise, we found it unnecessary to further translate weight/volume data into w/w data because this results anyway in a slight deviation toward the conservative direction (underestimation of exposure range). We agree that specialists may not perceive this as a good message, and if it were to be done over again we would probably proceed differently. But one should also keep in mind that experimental imprecisions might significantly outweigh rounding imprecisions, and therefore should be accounted for whenever possible. For instance, readers will notice from our methods (Supplementary data in Henry et al., 2012a) that our experimental dose (1.34 ng) was actually the result of a 34% excess dosage in our attempt to target a 1 ng dose. We could ascertain this 34% deviance because we ordered an expertise of our solution to ensure it was in the target range. We think that such an effort to remove an uncertainty level is rare enough to be worthy of note.

## Investigating context-dependent scenarios to deal with uncertainty

One option to deal with the uncertainty scaling issue is to move on from *worse case* scenarios toward *context-dependent* scenarios. This is at least how we may reformulate Guez's (2013) comment on temporal variations of oilseed rape nectar concentration. Risk assessors use the term *worse case* to describe exposure scenarios computed with the parameters upper bounds. Official conclusions on the peer review of the risk assessment may not always explicitly report worse case calculations (e.g., ANSES, 2012), with the underlying assumption that the conjunction of upper bound values in real field conditions would be exceptional—not to say unlikely. There is indeed a knowledge gap here. One should try and determine in which situations exposure parameters reach their upper bound, and whether the worse case combinations are likely to occur in real world. In other words, exposure levels in real world are context-dependent, and one should compute exposure scenarios with explicit reference to this context dependency. For instance, Guez (2013) computed *temporally explicit* thiamethoxam exposure scenarios, with regard to oilseed rape nectar concentration, and found that our dose actually respects the worse case scenario during a single flowering week out of four. He also investigated daylight time latitudinal variations with the underlying idea that a *spatially explicit* choice of the flight duration upper bound would help refine the exposure scenario. These are two nice examples of context-dependent scenarios with, respectively, a temporally and a spatially explicit adjustment. That said, the whole simulation remains to be updated using the recent advance in thiamethoxam residues measurements, and considering a complete range of latitudes (there was absolutely no reason why Guez should focus on the specific geographical locations of our study area). Spatially explicit adjustments may also consider temperature-dependent worse case flight durations or soil-dependent worse case nectar concentrations.

## Homing failure

We have emphasized here two new avenues of research, namely the uncertainty scaling issue and the context-dependency in pesticide risk assessment. But the greatest forthcoming challenge will be to lead this research throughout the future step forward into the biological levels, i.e., the model-based risk assessment at the colony scale (Osborne, 2012). The scale change from individuals to colony will require a rigorous management of uncertainty. This is the place to introduce our last, but not least, concern about Guez's comment. His criticism about the formula we used to investigate the consequences of homing failure from individuals to colony scale is misleading. What we need is a mortality *rate*, intended for use in the demographic models, not a *simple difference* between control and treated groups. The simple difference as an estimate of treatment-induced mortality is biased by the mortality due to the experiment *per se*. The difference must be expressed relatively to a reference value. To illustrate this, one can use voluntarily exaggerated values: imagine the studied dose causes half the individuals to fail homing under normal conditions (mortality due to homing failure = 0.5, this is what we want to estimate), but that on the same time, the experiment is so stressful that an overall 90% of control bees would fail homing. Then, homing success would be 10% for control groups and half less (5%) for treated groups. Based on these observations, the “simple” difference would return a much underestimated mortality rate due to homing failure [10–5% = 0.05], while our “relative” difference would return the appropriate rate [(10–5%)/10% = 0.5]. Think also about a scenario where 100% of exposed bees would fail homing. Then it would be impossible to get the proper mortality rate using a “simple” difference, by definition. In our example, the simple difference would return a 0.1 mortality due to homing failure instead of 1.

There is certainly place for improvement in the way to assess mortality due to homing failure, but this will be achievable providing the ecological dimension is properly implemented into classical toxicological approaches. In that respect, we fully support Guez's initiative to challenge our study. But unlike his concluding warning on the absence of empirical support for Rortais et al.'s (2005) and Khoury et al.'s (2011) methods, we would not refrain readers from exploring pesticide effects on honey bees using innovative tools.

## References

[B1] ANSES. (2012). Avis de l'Agence nationale de sécurité sanitaire de l'alimentation, de l'environnement et du travail relatif à une demande d'appui scientifique et technique dans la perspective de la publication de l'article “A common pesticide decreases foraging success and survival in honey bees”. Avis de l'Anses Saisine n° 2012-SA-0092.

[B2] Cresswell J. E. Thompson H. M. (2012). Comment on “A common pesticide decreases foraging success and survival in honey bees”. Science 337, 1453 10.1126/science.1224618 22997307

[B3] EFSA. (2012a). Statement on the findings in recent studies investigating sub-lethal effects in bees of some neonicotinoids in consideration of the uses currently authorised in Europe. EFSA J. 10:2752 10.2903/j.efsa.2012.2752

[B4] EFSA. (2012b). Scientific Opinion on the science behind the development of a risk assessment of plant protection products on bees (*Apis mellifera*, *Bombus* spp. and solitary bees). EFSA J. 10:2668 10.2903/j.efsa.2012.2668 PMC10173852 37179655

[B5] EFSA. (2013). Conclusion on the peer review of the pesticide risk assessment for bees for the active substance thiamethoxam. EFSA J. 11:3067 10.2903/j.efsa.2013.3067

[B6] Guez D. (2013). A common pesticide decreases foraging success and survival in honey bees: questioning the ecological relevance. Front. Physiol. 4:37 10.3389/fphys.2013.00037 23472059 PMC3590638

[B7] Henry M. Béguin M. Requier F. Rollin O. Odoux J.-F. Aupinel P. (2012a). A common pesticide decreases foraging success and survival in honey bees. Science 336, 348–350 10.1126/science.1215039 22461498

[B8] Henry M. Béguin M. Requier F. Rollin O. Odoux J.-F. Aupinel P. (2012b). Response to Comment on “A common pesticide decreases foraging success and survival in honey bees”. Science 337, 1453 10.1126/science.1224618 22461498

[B9] Khoury D. S. Myerscough M. R. Barron A. B. (2011). A quantitative model of honey bee colony population dynamics. PLoS ONE 6:e18491 10.1371/journal.pone.0018491 21533156 PMC3078911

[B10] Osborne J. L. (2012). Bumblebees and pesticides. Nature 491, 43–45 10.1038/nature11637 23086148

[B11] Rortais A. Arnold G. Halm M. P. Touffet-Briens F. (2005). Modes of honeybees exposure to systemic insecticides: estimated amounts of contaminated pollen and nectar consumed by different categories of bees. Apidologie 36, 71–83 16646488

